# Modulation of MicroRNAs and Exosomal MicroRNAs after Dietary Interventions for Obesity and Insulin Resistance: A Narrative Review

**DOI:** 10.3390/metabo13121190

**Published:** 2023-12-07

**Authors:** Karla G. Hernández-Gómez, Azalia Avila-Nava, Luis E. González-Salazar, Lilia G. Noriega, Aurora E. Serralde-Zúñiga, Rocio Guizar-Heredia, Isabel Medina-Vera, Ana Ligia Gutiérrez-Solis, Nimbe Torres, Armando R. Tovar, Martha Guevara-Cruz

**Affiliations:** 1 Departamento de Fisiología de la Nutrición, Instituto Nacional de Ciencias Médicas y Nutrición Salvador Zubirán, Mexico City 14080, Mexico; karla.hernandezg@incmnsz.mx (K.G.H.-G.); lilia.noriegal@incmnsz.mx (L.G.N.); xioheredia@gmail.com (R.G.-H.); nimbe.torrest@incmnsz.mx (N.T.); 2 Hospital Regional de Alta Especialidad de la Península de Yucatán, Mérida 97130, Mexico; azalia.avila@salud.gob.mx (A.A.-N.); ana.gutierrez@salud.gob.mx (A.L.G.-S.); 3 Servicio de Nutriología Clínica, Instituto Nacional de Ciencias Médicas y Nutrición Salvador Zubirán, Mexico City 14080, Mexico; luis.gonzalezs@incmnsz.mx (L.E.G.-S.); aurora.serraldez@incmnsz.mx (A.E.S.-Z.); 4 Departamento de Metodología de la Investigación, Instituto Nacional de Pediatría, Mexico City 04530, Mexico; imedinav@pediatria.gob.mx; 5 Escuela de Medicina y Ciencias de la Salud, Tecnológico de Monterrey, 14380 Mexico City, Mexico

**Keywords:** miRNA, exosomes, diet, obesity, insulin resistance

## Abstract

MicroRNAs (miRNAs) are small noncoding RNAs approximately 22 nucleotides in length. Their main function is to regulate gene expression at the posttranscriptional level by inhibiting the translation of messenger RNAs (mRNAs). miRNAs originate in the cell nucleus from specific genes, where they can perform their function. However, they can also be found in serum, plasma, or other body fluids travelling within vesicles called exosomes and/or bound to proteins or other particles such as lipoproteins. miRNAs can form complexes outside the cell where they are synthesized, mediating paracrine and endocrine communication between different tissues. In this way, they can modulate the gene expression and function of distal cells. It is known that the expression of miRNAs can be affected by multiple factors, such as the nutritional or pathological state of the individual, or even in conditions such as obesity, insulin resistance, or after any dietary intervention. In this review, we will analyse miRNAs whose expression and circulation are affected in conditions of obesity and insulin resistance, as well as the changes generated after a dietary intervention, with the purpose of identifying new possible biomarkers of early response to nutritional treatment in these conditions.

## 1. Introduction

MicroRNAs (miRNAs) are small RNAs of approximately 22 nucleotides in length that regulate gene expression at the posttranscriptional level by repressing target mRNAs. These small RNAs are responsible for the modulation of translation of most cellular proteins that were destined to be synthesized from target mRNAs, suggesting that miRNAs play an important role in the pathophysiology of many diseases [1]. The distribution and expression patterns of miRNAs are regulated by transcriptional and posttranscriptional mechanisms within the cell. Thus, miRNA profiling can be employed as a cellular identity due to the specificity of their expression in different tissues and conditions [2].

MicroRNAs can perform their function in the same cell where they are synthesized, or they can be taken to the extracellular space to travel in circulation, which constitutes one of the most recently studied cell-cell communication mechanisms in many physiological or pathological conditions.

Several studies have shown an association between the expression levels of different miRNAs and various pathologies, including obesity, type-2 diabetes, cardiovascular diseases, neurodegenerative disorders and cancer. It is also known that other factors, such as diet, exercise, or nutritional status, can modulate their expression [3].

A better understanding of how miRNA expression can be regulated by various pathologies and/or factors such as nutritional status and diet could contribute to the development of new therapeutic targets to control the balance of miRNAs within a cell that may prevent the development of metabolic diseases or be proposed as new biomarkers of disease to determine how subjects respond to different treatments, such as dietary treatments.

Therefore, the purpose of this review was to identify the circulating and cellular miRNAs with upregulated expression under conditions of obesity and insulin resistance that are reported in the literature, as well as the effects of different dietary interventions on their expression, to determine which miRNAs could play a role as biomarkers for early diagnosis, or to indicate response to treatments.

## 2. Biogenesis and Mechanism of Action of miRNAs

miRNAs synthesis begins in the nucleus through the expression of specific genes that code for MiRNAs. This process starts with RNA polymerase II, which synthesizes a primary miRNA also known as pri-miRNA. After transcription, the pri-miRNA sequence is processed by the complex formed by endoribonucleases called Drosha and DGCR8, resulting in a precursor miRNA sequence called pre-miRNA. This pre-miRNA is a sequence of approximately 60–80 nucleotides, which is exported from the nucleus to the cytoplasm by exportin 5 (XPO5) and Ran GTPases. Subsequently, the pre-miRNA is processed by the type-III endoribonuclease DICER and the RNA-binding proteins TRBP and PACT, giving rise to a double-stranded miRNA, which, after excision from the pre-miRNA hairpin conformation, becomes a mature miRNA [4]. Mature miRNAs then bind to their target mRNA through base-pairing within the RNA-induced silencing complexes (RISC), where mRNA stability and translation are regulated. miRNA-mediated RNA degradation can be achieved via a slicing-competent Argonaute-2 (AGO2) protein that cleaves the target mRNA in the mRNA and miRNA duplex. The function of RISC in translation control is mediated by GW182, which collaborates with AGO2 by recruiting deadenylation/decapping enzymes. This causes the unwinding of the duplex and the stable association of only one of the two strands. This guide strand directs target recognition through base pairing, while the other strand of the original small RNA duplex is discarded and represses translation [5,6].

As mentioned above, miRNAs may perform their function in the cell in which they are synthesized, or they may be present in body fluids or travel in the bloodstream to have a function in distal cells.

## 3. Circulating miRNAs: Novel Cell-Cell Communication

miRNAs can be exported and imported by cells through different mechanisms, one of the best known being within extracellular vesicles, bound to lipoproteins and carrier proteins, also known as protein-miRNA binding complexes. These are means of transport that make possible the communication of miRNAs with cells and tissues distal to where they were synthesized [7]. Due to this quality, miRNAs can be found in serum, plasma, urine, and other body fluids, making them the subject of recent studies as possible biomarkers in different diseases, since it is known that the amount and profile of miRNAs expressed in these transport media respond to different intracellular transcription factors and extracellular excretion stimuli.

### 3.1. Extracellular Vesicles: Exosomes

Exosomes are extracellular vesicles with a size < 200 nm originating from the Golgi apparatus. These components present a lipid bilayer due to their subsequent fusion of multivesicular bodies and the plasma membrane of the cell. Exosomes contain diverse molecules, such as RNAs, proteins, DNA fragments, and miRNAs, in different stages of maturation. The lipid bilayer protects those molecules from the extracellular medium during travel, and their degradation is more difficult, ensuring that most of the miRNAs inside them are delivered in good condition to the target cell [2]. Exosomes are detached from multivesicular bodies found in the cytoplasm and attached to the plasma membrane, where they fuse and are released by exocytosis [8]. Regarding what determines the cargo of these exosomes, studies suggest that there are different mechanisms involved in cargo and exosome recognition, such as ubiquitination and glycosylation of exosome membrane proteins, which couple the availability of miRNAs and other molecules within exosomes, the composition of exosomes varies significantly among different tissues and fluctuates based on the metabolic state of the cell [9].

It is known that the cellular release of exosomes also responds to diverse cellular stimuli, and unlike other means of transport of miRNAs, it seems to be a very well-orchestrated mechanism in different pathophysiological conditions or after pharmacological or dietary treatments. On the other hand, various mechanisms of exosome recognition with the target cell for their subsequent internalization have been described; some studies mention the recognition by antigen or membrane receptors of proteins found on the surface of exosomes such as the tetraspanins CD9, CD81, or the most abundant CD63, which also serve as anchors to the receptor cell [10].

Once bound to the surface of the new cell, exosomes can activate intracellular signalling pathways and discharge their contents into the cell by fusion to the membrane or enter the cell by phagocytosis, pinocytosis, or endocytosis [11].

Regarding their lifespan, studies investigating the uptake of exosomes in tissues have shown a half-life of exosomes from 2 to 60 min after being administered intravenously to animal models [12], suggesting not only rapid tissue signalling and communication after a stimulus but also an efficient mechanism of action of the miRNAs that reside in them.

### 3.2. miRNA-Binding Proteins and Lipoproteins

miRNAs can also be transported in the bloodstream by forming complexes with proteins, and like exosomes, they can enter the target cell through specific receptors for these proteins and release the miRNAs they transport to perform their function. It is suggested that this type of transport is only a fraction of the total number of circulating miRNAs. In miRNA-protein complexes, ribonucleoproteins, and nucleolar proteins such as nucleophosmin 1 (NPM1) have been found, which, once outside the cell, could also facilitate the loading of miRNAs into lipoproteins that also travel in the bloodstream [2]. In addition to exosomes and binding proteins, miRNAs can travel in smaller proportions bound to low-density lipoproteins (LDLs) and high-density lipoproteins (HDLs). HDL-bound miRNAs can be taken up by class B, type I scavenger receptors and subsequently released intracellularly to regulate gene expression. It has been shown that nutritional and metabolic status, e.g., hypercholesterolemia, can alter the abundance of lipoprotein-associated miRNAs, which can be totally distinct from those found within exosomes, thus indicating mechanisms of miRNA transport that are complementary and independent [13].

## 4. miRNAs in Obesity and Insulin Resistance

Several studies have explored changes in miRNA expression in different metabolic conditions, such as insulin resistance and obesity. Those studies utilized different research models to identify possible biomarkers for the prediction of type-2 diabetes and other cardiovascular diseases. Some of those studies also attempted to determine the response to miRNA treatments aimed at reversing that condition. Other studies have shown that miRNAs may be up- or downregulated in these conditions, and they have identified possible target genes that are influenced by those miRNAs, resulting in the regulation of the synthesis of proteins involved in the pathophysiological processes of obesity and insulin resistance (Table 1 and Table 2).

### 4.1. Obesity

Alterations in various circulating miRNAs have been found in human and animal models of obesity. Although most tissues contribute to the pool of circulating miRNAs, adipose tissue is known to play a key role. Alterations in adipose tissue, such as those occurring in various metabolic conditions, can cause significant changes in the expression of circulating miRNAs and thus negatively impact other tissues.

In human and animal models of obesity, an increase in circulating levels of microRNAs that regulate genes involved in metabolic conditions, such as energy expenditure or adipogenesis, has been found. A recent clinical study evaluated the effect of 4 different energy-restricted diets plus 90 min of moderate exercise per week on plasma miR-128-1-5p concentrations in overweight and obese subjects before and after 6 months. The results showed that a high level of miR-128-1-5p was associated with a higher HOMA-IR index (‘Homeostatic Model Assessment of Insulin Resistance’), waist circumference, and body fat percentage and a lower resting energy expenditure (REE), and an interaction analysis revealed that dietary fat and protein intake modify the relationship between the change in circulating levels of this miRNA and REE [28]. Regarding adipogenesis, a study in obese mice showed that after a high-fat diet, there was a significant increase in the exosomal miRNA miR-122, which regulated the expression of adipogenesis-related genes such as PPARγ (peroxisome proliferator-activated receptor γ), ADIPOQ (adiponectin), and the VDR/SREBF1 (vitamin D receptor/sterol regulatory element-binding transcription factor-1) axis, among others [20].

Growing interest exist in understanding how circulating miRNAs in exosomes participate in a very specific manner in the regulation of genes involved in the development of obesity and its comorbidities. One study showed that injection of exosomes containing miRNAs obtained from obese mice (miR-122, miR-192, miR-27a, miR-27b) into lean mice induced the development of insulin resistance and dyslipidaemia, suggesting that these miRNAs play an important role in the pathophysiology of obesity, the development of type-2 diabetes, and possibly other cardiovascular diseases [26]. The conception of microRNAs as possible biomarkers of cardiovascular disease in the presence of obesity has been proposed by different research groups worldwide. A cross-sectional study conducted in adolescents with obesity found an association between circulating levels of at least 10 microRNAs and plasma levels of adipokines such as adiponectin, leptin, and other markers of metabolic syndrome such as glucose, insulin, the HOMA-IR index, peptide-C, and plasma levels of lipids such as triglycerides, HDL cholesterol, and LDL cholesterol [32].

On the other hand, it is important to consider that obesity is a condition in which a proinflammatory state prevails, and this chronic inflammatory environment in turn favours the appearance of various cardiovascular complications, including atherogenesis. Tang and coworkers show that exosomal miR-27b-3p, derived from visceral adipocytes, is involved in endothelial inflammation and atherosclerosis. Mechanistically, miR-27b-3p binds directly to the protein-coding sequence (CDS) region of PPARα mRNA, thereby decreasing the protein expression [14]. Also, Zhang and coworkers evaluated one of the mechanisms involved in the proinflammatory environment orchestrated by macrophages during obesity in an animal model and in vitro, and they found that exosomal miR-1224 participates in the inhibition of M2 macrophages in adipose tissue [18]. Another study that evaluated the conditions that promote an anti-inflammatory environment regulated by M2 macrophages found that exosomes obtained from THP-1 macrophages polarized with IL-4 (anti-inflammatory interleukin) promoted the expression of miR-21, miR-99a, miR-146b, and miR-378a while decreasing miR-33 expression, and it was also found that there was increased expression of PPARγ and GLUT4 (glucose transporter type 4), improving glucose uptake and insulin metabolism, increasing UCP1 (uncoupling protein 1) and OXPHOS (oxidative phosphorylation system) activity, and promoting lipophagy, mitochondrial activity and beiging in adipocytes [21]. Interestingly, it has been shown that M2 macrophages with anti-inflammatory effects are able to release exosomes with miRNAs that also exert an anti-inflammatory function, as is the case for miR-690, which directly regulates NADK (nicotinamide adenine dinucleotide kinase). NADK modulates macrophage-mediated inflammation and participates in insulin signalling as a gene encoding NAD+ kinase, an enzyme that converts nicotinamide adenine dinucleotide (NAD+) to NADP+. The inhibitory effects of NAD+ kinase on cellular insulin action were evidenced by improved in vitro insulin signalling after siRNA-induced depletion of NADK. Previous studies have shown that during obesity, NAD^+^ levels are reduced through either repression of NAD+ biosynthesis or greater NAD^+^ utilization. The authors suggested that miR-690 could even become a new insulin sensitizing agent for the treatment of metabolic diseases [22]. The miR-34a released from adipose tissue exosomes inhibits polarization to M2 macrophages possibly by repressing the expression of KLF4 (Krueppel-like factor 4), a transcription factor involved in stem cell differentiation plasticity, thereby promoting fat tissue-induced inflammation in obesity, and the researchers found that miR-34a expression increases proportionally with the development of diet-induced obesity [25].

Other miRNAs that could also participate in the pathophysiology of obesity-related hepatic steatosis have been studied. Castaño and coworkers [19] studied the regulation of the expression of local miRNAs and circulating exosomal miRNAs in an obesity model induced by a high-fat or high-sucrose diet, these miRNAs were found to be associated with the progression of obesity and the regulation of hepatic steatosis related genes, such as PTGDS (prostaglandin D2 synthase), GGT1 (gamma-glutamyltransferase 1), HK3 (hexokinase 3), PFKP (phosphofructokinase), PKM2 (pyruvate kinase M2), SLC2A5 (solute carrier family 2 member 5), and G6PC (glucose-6-phosphatase catalase). These genes are involved in metabolic pathways such as gluconeogenesis, de novo lipogenesis and insulin signalling. Another research group used an animal model and in vitro studies to analyse how Let-7b-5p, a miRNA, could regulate genes such as UCP1, FATP1 (fatty acid transport protein-1), ATP5A (ATP synthase α-subunit), PGC-1α (peroxisome proliferator-activated receptor γ coactivator 1α) for selfregulation of thermogenesis and oxidative phosphorylation, as well as participate in the hepatic TGF-β (transforming growth factor-beta) signalling pathway and inhibit white adipose tissue beiging, which could also promote the development of non-alcoholic fatty liver disease and obesity [17]. A cross-sectional clinical trial conducted in adolescents compared the expression profile of circulating exosomal miRNAs in the serum of those with obesity and those with non-alcoholic fatty liver disease. This study showed a significant increase in three miRNAs in the group of adolescents with non-alcoholic fatty liver disease (miR-122-5p, miR-27a, miR-335-5p), which have been identified as possible biomarkers of this disease. These miRNAs regulate genes such as WNT10B (Wnt family member 10B), PPARγ and SREBP-1c (sterol regulatory element-binding protein-1c), which are involved in hepatic lipid metabolism and adipocyte differentiation [30].

A study in an animal model of hepatic steatosis induced by a high-fat diet evaluated the effect of different interventions, such as a low-fat diet, energy restriction, quercetin supplementation and exercise, on miRNAs that act to regulate the synthesis and action of thyroid hormones. The study showed that although the four interventions improved hepatic steatosis to different degrees, energy restriction showed the greatest improvement in the reduction of steatosis, as well as a possible upregulation of NIS (iodine transporter protein) expression mediated by miRNAs miR-200a, miR-28, miR-339, miR-383, and miR-146b. All four interventions increased thyroid hormone action, possibly by differentially regulating miRNAs that upregulate TRB (T-cell receptor beta locus) and DIO1 (iodothyronine deiodinase 1) expression and by maintaining the homeostasis status dependent on Nrf2 (Nuclear factor erythroid 2-related factor 2), a transcription factor involved in redox homeostasis and antioxidant gene expression [23].

Other studies have sought to identify the abilities of adipose tissue exosomal microRNAs in models of obesity, including cellular repair [31]. An in vitro study of mesenchymal stem cells taken from adipose tissue in subjects with and without obesity demonstrated how exosomes isolated from those cells have the capacity to modulate inflammation, apoptosis processes, and the activation of the MAPK (mitogen-activated protein kinase) and Wnt signalling pathways involved in these processes. Moreover, some miRNAs inside those exosomes, such as miR-1291, miR-888-5p, miR-6892, miR-222-5p, miR-8072, miR-4757-5p, miR-769-5p, and miR-4730, were found to be overexpressed in the cells of the participants with obesity (Table 1).

### 4.2. Insulin Resistance

The persistent inflammatory state present in obesity is closely linked to the development of insulin resistance, and the mechanisms involved in this condition have been studied in various populations. A cross-sectional clinical trial conducted in adolescents with and without obesity aimed to compare plasma levels of exosomal miRNAs, and the results showed that there was a decrease in miR-223-5p, 33a-3p, miR-181a-5p, and miR-199-5p in those who had obesity. These miRNAs regulate genes such as CHD9 (chromodomain helicase DNA binding protein 9), PTEN (phosphatase and tensin homologue), MTMR12 (myotubularin related protein 12), TBL1X (transducin beta-like 1 X-linked), and CPOX (coproporphyrinogen oxidase), which are involved in lipid metabolism, inflammatory status and insulin resistance, suggesting these miRNAs as biomarkers of obesity in adolescents [29]. The levels of miR-27a and its correlation with insulin resistance were assessed within a pediatric population, and the results showed that serum miR-27a levels were positively correlated with obesity and insulin resistance in children with obesity. This same study also used an animal model with C57BL/6J mice with diet-induced obesity and C2C12 cells and found that in addition, incubation of miR-27a obtained from adipocytes of mice with induced insulin resistance caused insulin resistance in C2C12 skeletal muscle cells. Furthermore, they found that miR-27a suppressed the expression of PPARγ, i.e., genes involved in the development of obesity [60].

Inflammation caused by obesity leads to alterations in insulin signalling pathways. Ying et al. [16] demonstrated in vitro and in an animal model how adipose tissue releases into the bloodstream exosomes with microRNAs that travel to insulin-sensitive tissues such as liver and muscle, as mice with obesity treated with exosomes from adipose tissue of lean mice, with a higher proportion of M2 macrophages, decreased insulin resistance. They also found an increase in miR-155 in adipose tissue exosomes from obese models, which could contribute to the development of insulin resistance.

Another study in an animal model showed that mice with obesity and fed a high-fat diet had lower levels of exosomal miR-141-3p than mice with obesity consuming a normal diet, and the decrease in miR-141-3p was associated with reduced Akt (serine/threonine-protein kinase) phosphorylation. They also found that GW4869, a protein involved in the inhibition of exosome biogenesis and release, might participate in the mechanism of action of miR-141-3p in hepatocytes, which is expressed in higher proportions in models of obesity [24].

In the search to identify microRNAs that may be useful as diagnostic biomarkers and therapeutic targets, several research groups have evaluated how their expression is regulated in the presence of insulin resistance. Ali and collaborators studied the serum levels of miRNAs, mRNAs, and lncRNAs (long noncoding RNAs) in subjects with type-2 diabetes, subjects with prediabetes, and healthy control subjects, with the purpose of identifying genes broadly related to the pathogenesis of insulin resistance and prediabetes. The results showed that the expression levels of TMEM173 (Transmembrane Protein 173), CHUK (Conserved Helix-loop-helix Ubiquitous Kinase), and the miRNAs miR-611, miR-5192, and miR-1976 gradually increased from the control group to the prediabetes group and reached their highest levels in the type-2 diabetes group, while the expression of lncRNAs RP4-60503.4 and AC074117.2 gradually decreased from the control group to the prediabetes group and reached their lowest levels in the type-2 diabetes group [55].

In a cross-sectional clinical study, results revealed that women with metabolic syndrome showed decreased levels of miR-16 and miR-363, while men with one or more risk factors displayed elevated levels of miR-Let-7c and miR-30a. Moreover, there was a positive correlation between waist circumference and higher levels of miR-Let-7c, miR-122, miR-30a, miR-146a, miR-15a, miR-30d, and miR-222. Additionally, individuals with higher plasma glucose and/or greater insulin resistance exhibited increased expression of miR-122, miR-139, miR-Let-7c, miR-126, and miR-30a [56].

Insulin resistance may be related to the development of multiple complications in different insulin-sensitive tissues. In fact, an in vitro study showed the mechanism by which adipose tissue can regulate cardiac insulin resistance through miRNAs. For this purpose, exosomes were extracted from 3T3-L adipocytes that were induced to insulin resistance and used to treat rat ventricular myocytes. The findings were that miR-802-5p was highly expressed in models with insulin resistance and that heat shock protein 60 (HSP60) was its target gene. Its depletion was accompanied by a significant increase in C/EBP (CCAAT/enhancer binding protein) expression and increased phosphorylation of JNK (c-Jun N-terminal kinase) and IRS1 (insulin receptor substrate-1) on serine 307. Therefore, exosomal miR-802-5p from hypertrophic adipocytes caused cardiac insulin resistance through downregulation of HSP60 [41] (Table 2).

## 5. Regulation of miRNAs by Diet

miRNAs have been proposed as biomarkers of disease, which could be regulated in response to strategies and/or treatments for some pathologies. One of these alternatives is dietary changes, which may be a fundamental treatment component, especially in metabolic diseases. Studies have evaluated changes in miRNAs after a nutritional intervention to determine the effect of nutrients, supplements, foods, dietary patterns, or energy restriction in the diet.

### 5.1. Energy Restriction

Energy restriction is one of the most widely explored dietary interventions to assess the difference in the expression of various miRNAs that may be altered under conditions of obesity in both animal models and humans.

A recent study evaluated the difference in miRNA expression in rhesus monkeys after prolonged caloric restriction. The study found changes in the expression of 24 known miRNAs and 10 novel miRNAs not known until then. They showed correlations between body weight, adiposity and insulin sensitivity, with at least 10 miRNAs upregulated after dietary energy restriction. miR-125a-5p presented the greatest decrease in its expression after the intervention; in addition, a positive correlation was found between miR-125a-5p abundance and adiposity and a negative correlation with insulin sensitivity [61]. Other microRNAs that decreased in expression after intervention were miR-16, miR-20a, miR-21, miR-92a-5p, miR-130 a-5p, miR-143-5p, and miR-224. It is known that miR-92a-5p is synthesized in brown adipose tissue, a metabolically active tissue for energy expenditure, and has been inversely correlated with brown fat activity in humans [62]. Another study in an animal model found that after 4 weeks of intervention, mice fed an energy-restricted diet had a significant decrease in CD8+ T cells as well as an increase in serum miR-16-5p, miR-196b-5p and miR-218-5p expression compared to the control group. Subsequently, miR-16-5p was shown to decrease the mRNA expression of the proinflammatory cytokines IL-1β, IL-6, and TNFα, which have been implicated in the dysregulation of the insulin signalling pathway [63]. Specifically, it has been reported that TNFα inhibits the proper phosphorylation of IRS-1, favouring the development of insulin resistance. This study suggests that miR-16-5p plays an important role in the anti-inflammatory regulation conferred by a low-energy diet, as well as indirectly enhancing insulin signalling [64].

In addition to interventions, it has been shown that rapid weight loss can affect the regulation of microRNA expression. In fact, a recent study with obese subjects showed that after a severely energy-restricted diet (<800 Kcal/day) for 4 weeks, there was a modulation in circulating levels of 75 miRNAs. Most notably, miR-34a, miR-208, miR-193, miR-320, miR-433, miR-568, and miR-181a have been proposed as biomarkers of beneficial effects in response to weight loss in individuals with obesity [65].

### 5.2. High-Protein Diet

An increase in protein intake has been associated with beneficial effects on pathologies such as excess weight. There is now evidence that these benefits may be associated with the regulation of miRNAs. A study showed that men with obesity who consumed a high-protein diet (30% of energy intake) during a 12-week weight-loss intervention had a decreasing trend in circulating levels of miR-223 [36]. This miRNA belongs to a subgroup of miRNAs that travel in the circulation bound to high-density lipoproteins (HDLs). As mentioned above, miR-223 has been found to be increased in populations with obesity and insulin resistance; furthermore, this miRNA was found to modulate the inflammatory phenotype and macrophage activation through the FBXW7/TLR4 (F-box and WD repeat domain containing 7/toll-like receptor 4) axis. FBXW7 is one of the proteins of the ubiquitin ligase complex, and it functions in ubiquitination recognition. Recently, the role of FBXW7 in attenuating TLR4 signalling processes in macrophages has been demonstrated [66].

A study involving men older than 70 years (*n* = 31) and without any diagnosed disease who consumed a high-protein diet (1.6 g/kg/d) for 10 weeks revealed decreased expression of 5 miRNAs (125b-5p, miR-100-5p, miR99a-5p, miR-23b-3p and mir-203a) compared to the normal-protein diet group (0.8 g/kg/d). These miRNAs have been reported to be gene regulators of proteins involved in various proinflammatory cascades, such as interleukin 6/signal transducer and activator of transcription 3 (IL6/STAT3). The authors propose that a high-protein diet may regulate proinflammatory responses [67].

### 5.3. Ketogenic Diet

In addition, the regulation of energy and protein intake has been used as a strategy for weight loss or to reduce metabolic disturbances. Additionally, diets high in lipids and low in carbohydrates have been reported as a strategy with beneficial effects on metabolic pathologies. A clinical study in obese subjects (18 women and 18 men) evaluated the effect of a 6-week two-step ketogenic diet on body composition and microRNA expression profile in obese subjects. The results showed significant changes in the expression of three miRNAs, miR-Let-7b-5p, miR-143-3p, and miR-504-5p, all related to the regulation of genes linked to nutrient metabolism, such as mTOR (mechanistic target of rapamycin), PPARs (peroxisome proliferator-activated receptors), insulin and proinflammatory cytokine signalling pathways [68].

Cannataro and coworkers evaluated the expression profile of microRNAs in patients with obesity in response to treatment with a ketogenic diet. After consumption of the ketogenic diet, there was a significant upregulation in the expression of miRNAs compared to subjects with obesity who did not consume the diet. In addition, it was observed that the expression of miRNAs let-7b-5, miR-143-3p, miR-148b-3p, miR-590-5p, miR-520 h, miR-644a, and miR-548d-3p in obese subjects was similar to that in lean subjects. Interestingly, bioinformatic analysis showed that 4 miRNAs are linked to antioxidant and anti-inflammatory metabolic pathways, such as Let-7e-5p, which regulates glutathione peroxidase 7 (GPX7) synthesis, miR-520 h, which regulates Tet methylcytosine dioxygenase 3 (TET3), and miR-548d-3p, which is related to the regulation of superoxide dismutase 2 (SOD2), while miR-30a-5p correlated with decreased catalase expression in red blood cells. The authors concluded that the ketogenic diet results in similar regulation of miRNA expression in obese subjects as in lean subjects [33].

### 5.4. Diet with Supplementation

Dietary supplementation has been utilized to combat obesity, as it can contribute to weight loss and attenuate some related risks, even without changes in macronutrient intake. Due to the changes associated with these types of interventions, there are studies that suggest the possible role of miRNAs in these benefits. A randomized, crossover clinical study in subjects with prediabetes (*n* = 49) evaluated the effect of a pistachio-enriched diet on the expression profile of microRNAs related to glucose metabolism and insulin resistance. Participants underwent 2 conditions, a pistachio-supplemented diet (57 g/day) and an isocaloric control diet, for 4 months with a 2-week washout period. Pistachio supplementation increased the expression of miR-21, miR-29b, miR-223, and 15a, which was reflected in a decrease in insulin resistance. Subjects who increased the levels of miRNAs 15a and 21 also showed a decrease in IL-6 expression, which may result in a decrease in the inflammatory state [69]. Specifically, miR-223 has been reported to induce the expression of GLUT4 protein to normalize glucose levels [70]. miR-15a regulates insulin biosynthesis by binding to UCP-2 in mice [71]. For miR-21, a previous study showed that this miRNA reversed insulin resistance in 3T3-L1 cells and significantly increased insulin-induced glucose uptake by translocating GLUT4 to the cell membrane [72]. It was also observed that pistachio supplementation decreased the levels of miR-375 and miR-192. It has been suggested that increased levels of miR-375 indicate increased expression from its synthesis in pancreatic islets in subjects with type-2 diabetes, prediabetes, and alterations in glucose or insulin [73]. miR-192 has been reported to be elevated in subjects with prediabetes and in animal models of mice with glucose intolerance [61]. Thus, pistachio consumption in these amounts could regulate some metabolic pathways involved in glucose metabolism by action of these miRNAs.

In an animal model, it was shown that a diet supplemented with soy (25%) for 28 days promoted significant changes in the expression of two miRNAs, miR-145a-5p and miR-455p, compared to the control group. The analysis suggests that these miRNAs may impact nuclear receptor expression, the glutamine/glutathione ratio, catalytic activity and iron metabolism in the liver [74]. Another study evaluated the effect of a diet high in protein, fish oil and omega-3 in NZ10 mice, which are predisposed to develop obesity and type-2 diabetes. After 19 weeks of intervention, an increase in the expression of miR-205 and a decrease in the expression of miR-411, miR-155, miR-335, and miR-21 were observed compared to the control group (<6% fat). Increase levels of these miRNAs have been reported to be involved in the progression of type-2 diabetes or hepatic steatosis, suggesting that polyunsaturated fatty acids such as omega-3 may regulate gene expression at the hepatic level. Other studies have shown that these miRNAs improve insulin sensitivity and decrease liver triglycerides and hepatic steatosis in animal models [75]. In an animal model, the effect of fish oil supplementation on the hepatic expression of miRNAs and its relationship with the development of non-alcoholic fatty liver disease were evaluated. Separate male Sprague-Dawley rats were included in 3 intervention groups: one group was fed a control diet, the second group was fed a high-fat, high-cholesterol diet, and the third group was fed a high-fat, high-cholesterol diet plus fish oil supplementation. The expression of microRNAs in liver tissue was analysed. The analysis showed that there was a difference in the expression of 79 miRNAs between the high-fat diet with fish oil supplementation and the high-fat diet without fish oil supplementation. The miRNAs that were differentially expressed included miR-29c-3p, miR-30d-5p, miR-33-5p, miR-34a, and miR-328a-3p. It has been reported that these miRNAs might interact with hepatic lipid metabolism, as well as important transductional regulation of different genes, such as Pcsk9 (proprotein convertase subtilisin/kexin type 9), Insig2 (insulin-induced gene 2), Per3 (period circadian regulator 3), and Socs1/3 (suppressor of cytokine signalling 1 y 3), which are related to the development of non-alcoholic liver disease [76].

### 5.5. Other Related Interventions

A recent review aimed to identify the effect of physical training on miRNAs associated with systemic arterial hypertension, type-2 diabetes, and obesity. It describes the increased expression of 8 miRNAs, including miR-21, miR-27a, miR-30d, miR-126, miR-181a, miR-143, miR-222, and miR-223. These miRNAs have been found to participate in the regulation of genes involved in mechanisms of insulin resistance, adipogenesis or angiogenesis, suggesting a regulatory mechanism with metabolic benefits of physical activation [77].

The effects of an exercise program combined with a weight-loss diet in adolescents with obesity have also been evaluated. This program was shown to decrease glucose levels and circulating levels of miR-126, which has been correlated with parameters of microvascular function [34]. miR-126 has also been linked to IRS1 and to mitochondrial dysfunction, causing altered signalling in the insulin pathway and decreased glycogen synthesis [78].

A recent clinical trial analysed the effects of weight-loss bypass surgery on the upregulation of the expression of microRNAs present in circulating exosomes of six African-American women with obesity (BMI 51 ± 8.8 kg/m^2^). One year after surgery, there was a decrease in BMI (−18.6 ± 5.1 kg/m^2^), HOMA index (1.94 ± 0.6 to 0.49 ± 0.1) and concentration of branched-chain amino acids. In addition, upregulation was found in the expression of 168 microRNAs, of which 10 were directly linked to the regulation of genes involved in the insulin signalling pathway: miR-155-5p, miR-503-5p, miR-199a-5p, miR-539-5p, miR-874-3p, miR-4664-5p, miR-4747-5p, miR-516b-5p, miR-126-3p, and miR-122-5p [79].

## 6. miRNAs Related to Obesity, Insulin Resistance and Diet

Obesity is a condition that is usually accompanied by a long list of metabolic ailments, including insulin resistance. Recent studies have estimated that up to 1 in 2 people with obesity have insulin resistance. Insulin resistance is the precursor to the development of type-2 diabetes, which coexists with the presence of cardiovascular disease. The first line of treatment for both obesity and obesity-related insulin resistance is to make dietary changes with the goal of pursuing healthy weight loss, where diet is a central part of the treatment of these patients. As discussed in this review, both obesity and insulin resistance can be identified through the regulation of characteristic miRNAs, and interestingly, after dietary intervention, we seek to reverse the expression profile of these miRNAs.

**Figure 1:**
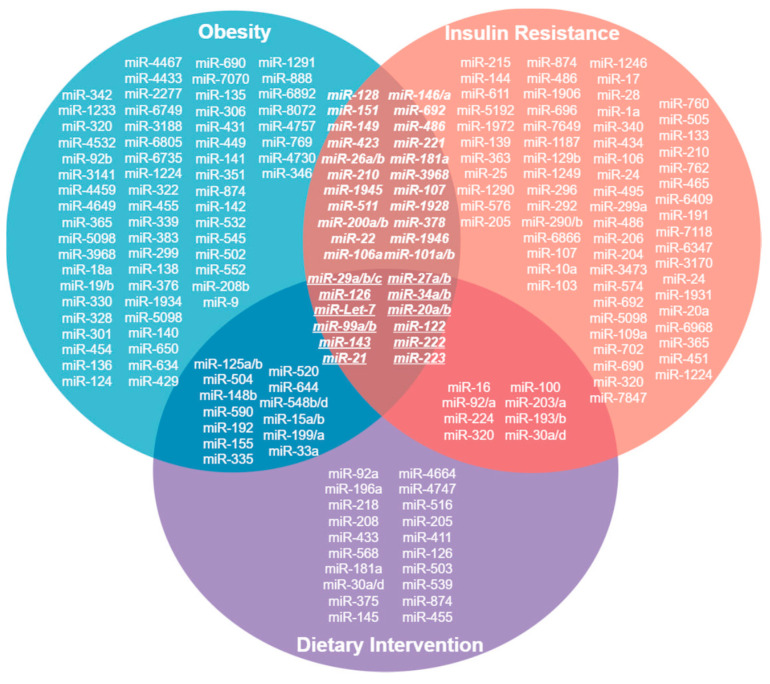
Venn Diagram. MicroRNAs whose expression is upregulated in conditions of obesity, insulin resistance and after a dietary intervention. 12 of them converge in these conditions and could be proposed as biomarkers of early response in the dietary treatment of obesity and insulin resistance.

We identified miRNAs that are up- and downregulated in conditions of obesity and insulin resistance, and after dietary intervention, these could be proposed as possible biomarkers of early response to nutritional treatment of obesity and insulin resistance (Figure 1) (Venn diagram).

## 7. Discussion

This review summarizes some of the changes in miRNA expression under certain pathologies associated with chronic degenerative diseases. Interestingly, some miRNAs that are upregulated after dietary intervention in conditions of obesity and insulin resistance have been identified (Figure 1). These miRNAs could be proposed as possible biomarkers of early response to nutritional treatment of obesity and insulin resistance. Therefore, it is of great importance to understand the different expression profiles of microRNAs in different pathologies in order to know what is occurring at the cellular level, from the regulation of proteins involved in different signalling pathways to the regulation of proteins that play a decisive role in the development of diseases and their complications. Thus, miRNAs may not only be biomarkers of disease stages but also prognostic biomarkers or biomarkers of response to treatments aimed at preventing or reversing these pathophysiological mechanisms in a timely manner.

This literature review included 26 recent articles that extensively studied the expression of microRNAs in models of obesity in animals and humans. Thus, there is no doubt that there is a wide difference between the circulating levels of microRNAs in obese subjects compared with nonobese subjects, supporting the theory that the disease condition is able to modulate the expression of miRNAs and that their identification in the circulation is an excellent alternative to know what is occurring systemically in various organs. As we could observe in this review, chronic and persistent inflammation, as well as the metabolic consequences that this causes, is one of the most widely studied conditions in models of obesity, and the regulation of genes such as NADK and KLF4, which are mediated by miRNAs such as miR-690 and miR-34a, suggests that microRNAs are directly involved in the polarization of macrophages to an anti-inflammatory or proinflammatory phenotype [22,25].

Studies on the regulation of miRNAs in insulin resistance, which is a pathology closely related to obesity, were also included. Twenty-two articles studying how microRNAs modulate their expression in the presence and development of insulin resistance were included in this review. Animal model and in vitro studies described how miRNAs alter insulin signalling pathways at different times. For example, increased levels of circulating miR-222 were related to the presence of insulin resistance as well as decreased expression of IRS1 in adipose tissue [50].

miRNAs are not only proposed as biomarkers of disease state but also as biomarkers in response to treatment, suggesting the need to search for a suitable biomarker for clinical practice. In this sense, it is important to remember that one quality of biomarkers is that they must be noninvasive, so considering circulating miRNAs, especially in blood, seems to be the best alternative; of those circulating miRNAs, those travelling inside exosomes are the best option, since as mentioned above, exosomes confer protection to miRNAs due to their lipid bilayer, making their travel in the bloodstream safe to ensure their effect on target cells. In addition, according to the literature, research aimed at studying exosomal miRNAs in various pathologies, such as cancer, has grown exponentially, as researchers have sought to elucidate their performance as a biomarker and even as a treatment vehicle.

Interestingly, the regulation of microRNA expression is a reversible epigenetic process that is sensitive to the pathophysiological state of the individual and has also demonstrated its response to nutritional interventions in the different study models, which leads us to believe that the basal nutritional state of the subjects may also be a factor in the regulation of the expression of these miRNAs. Unfortunately, there are very few published studies that discuss the impact of a specific nutritional treatment on the modulation of miRNA expression. In this review, we found only 12 studies that evaluate how diet changes the abundance of miRNAs after a period of treatment compared to baseline levels, and of these, only six are clinical trials conducted in humans.

As shown in this review, there are still few clinical studies in humans that seek to find differences in the expression of miRNAs in metabolic conditions, such as obesity and insulin resistance, together with a particular nutritional intervention. This represents not only a remaining challenge for researchers in the field of nutrigenomics but also a window of opportunity for the innovation of biomarkers of early response to nutritional treatments, which will allow the proposal of new effective strategies for the comprehensive management of patients with cardiovascular diseases, which are the leading causes of death in Mexico and the world.

The possibility of understanding metabolic conditions from the regulation of gene expression makes the study of microRNAs an opportunity to address the future of disease diagnosis, treatment and control.

## Figures and Tables

**Table 1:** MiRNAs, exosomal miRNAs, obesity, and diet.

Author	Study Population	Study Design	Dietary Intervention/Treatment	miRNAs Regulated	Significant Target Genes	Metabolic Effect	Tissue	Exosomes
In vitro studies								
Tang et al. [14]	Gonadal adipocytes from WT and ob/ob mice Human umbilical vein endothelial cells ApoE-/- mice.	In vitro study.	In animal model WT and ob/ob mice: Group 1. Normal diet Group 2. High-fat diet. In vitro model: TNF-α stimulation. In animal model ApoE-/- mice: Western diet.	↑ miR-221, miR-22, miR-27b, miR-27a, miR-342, miR-34a-5p, miR-101b-3p, miR-22-3p, miR-222-3p	↓ VCAM1, ICAM1, MCP1, P65, PPARα	Proinflammatory pathways Endothelial inflammation Adipogenesis Atherogenesis.	Cell culture supernatant Serum.	Yes
Aas et al. [15]	Human myotubes with obesity (BMI 45 ± 5 kg/m^2^) and diabetes (HbA1c 6.7 ± 1.2%).	In vitro study.	Electro pulsed stimulation.	↑ miR-1233-5p, miR-320b, miR4532, miR-92b-5p, miR-3141, miR-4459, miR-4649-5p, miR-4467, miR-4433b-3p, miR-2277-5p, miR-6749-5p, miR-3188, miR-6805-5p, miR6735-5p	↑ GANAB, VCL, SYNPO2, TFRC, WARS, PLEC, EEF2, MAP4, SND1, RPS15A, ZYX, CNDP2, MAP1A, RNH1, DES, GSN, FAM129B, PLOD3, AARS ↓ S100A7, COL6A1, PVR, CLSTN1, SEMA7A, RERPING1, CSF1, BMP1, APOE, SDF4	Receptors of the serine/threonine protein kinase pathway Ubiquitin proteasome system Guanine nucleotide exchange factor.	Cell culture supernatant.	Yes
Ying et al. [16]	3T3-L1 adipocytes WT mice with obesity WT mice without obesity Primary hepatocytes of WT mice Primary myocytes of WT mice.	In vitro study Animal model.	Stimulus: miR-223 Obese mice: High-fat diet. Nonobese mice: Normal diet + adipose tissue exosomal miRNAs Stimulus with glucagon and insulin.	↑ miR-223, miR-155, miR-181, miR-149, miR-210, miR-1945 ↓ miR-3968, miR-692, miR-365, miR-7070, miR-692, miR-5098, miR-1928, miR-690, miR-7054, miR-682, miR-1946, miR-511	↓ PPARγ, GLUT4, ERα, AKT	Insulin signaling pathway.	Cell culture supernatant.	Yes
In vivo studies in animal models								
Zhao et al. [17]	TGF-β receptor II (Tgfbr2)-deficient C57BL/6 mice C57BL/6 mice expressing TGF-β receptor II (Tgfbr2 flox/flox) AML-12 cell line Humans with obesity (BMI ≥ 30) and hepatic steatosis Nonobese and healthy humans.	Animal model In vitro study Cross-sectional clinical study.	High-fat diet (60% of L) both groups of mice Normal chow diet (10.2% of L) both groups of mice 4 groups at the end 16 weeks 2 more groups on β3-AR agonist stimulation 1 mg/kg/day 7 days 2 more groups with exposure to temperature of 4 °C.	↑ Let-7b-5p	↓ CD36, FATP1, FABP1, ATP5A, COX5B, UCP1, DIO2, PRDM16, PGC-1A	Thermogenesis Mitochondrial oxidative phosphorylation.	Cell culture supernatant Serum.	Yes
Zhang et al. [18]	Mice with obesity Mice without obesity KO mice for miR-1224 Primary adipocytes.	Animal model In vitro study.	Animal model: High-fat diet Standard diet.	↑ miR-1224	↓ MSI2	Wnt/β-catenin pathway Inhibition of M2 macrophage polarization Inflammation.	Plasma Cell culture supernatant.	Yes
Castaño et al. [19]	Mice with obesity Mice with hepatic steatosis Mice without obesity or hepatic steatosis.	Animal model.	Model with obesity: High-fat diet Model with hepatic steatosis: Normal diet Chow + sucrose in water Control model: Normal diet Chow 15 weeks.	↑ miR-22-3p, miR-122-5p, miR-223-3p, miR-34a-5p, miR-378a-3p, miR-101-3p, miR-107-3p, miR-19-3p, miR-200b-3p, miR-322-5p ↓ miR-199a-5p, miR-23b-3p, miR-143-3p	↓ PTGDS, GGT1, HK3, PLKP, GM13882, PKM2, GOT1, DLD, G6PC, ENO3 ↑ SLC2A5, GPI1, ALDOC,	Hepatic steatosis Gluconeogenesis De novo lipogenesis Insulin resistance.	Serum Hepatocytes.	Yes
Huang et al. [20]	Mice with obesity.	Animal model.	High-fat diet.	↑ miR-122	↓ VDR, SREBF1, PPARγ, ADIPOQ, LPL	Adipogenesis.	Cell culture supernatant	Yes
Phu et al. [21]	Obese mice Human THP1 monocytes 3T3-L1 adipocytes WT mice with obesity Ldlr-/- mice.	Animal model In vitro study.	High-fat diet 6 weeks In WT mice with obesity and Ldlr-/-: Intraperitoneal THP1-IL-4 exosomes.	↑ miR-21, miR-99a, miR-146b, miR-378a ↓ miR-33	↑ PPARγ, GLUT4, UCP1, OXPHOS	Lipophagy Mitochondrial activity Oxidative phosphorylation Beiging Hepatic steatosis Glucose metabolism Insulin resistance.	Plasma Cell culture supernatant.	Yes
Ying et al. [22]	Mice with obesity 3T3-L1 adipocytes L6 myotubes Primary hepatocytes.	Animal model In vitro study.	High-fat diet 8 weeks + treatment with M2-type macrophage exosomes 4 weeks.	↑ miR-690, miR-7070, miR-365, miR-5098, miR-3968	↓ NADK, BMDM, RAW264, PPARγ, YM1, MRC1, INOS, TNFA. IL1B, CC12, IFBG	Citrate cycle Pyruvate metabolism Glycolysis Gluconeogenesis JAK STAT pathway signaling Insulin pathway signaling mTOR pathway signaling.	Cell culture supernatant.	Yes
Xia et al. [23]	C57BL/6J Mice.	Animal model.	High-fat diet for 10 weeks, then divided into 5 groups: High-fat diet Low fat diet (10% L) Energy-restricted diet (low-fat diet with 70% energy restriction) Quercetin-enriched diet (0.005% quercetin) Diet with exercise For 7 weeks.	Liver: ↑ miR-28, miR-382, miR-146 Thyroid: ↓ miR-200a, miR-339, miR-383, miR-146	↑ TRB, SREBP1C, FASN, ACC1, SCD1, CD36, FABP, G6PC, PCK1, FAGF21, PGC1α ↓ NIS, NRF2, NQO1, HO-1, DIO1, ATP5C1, COX7C	Energy metabolism Lipid metabolism Thyroid gland function.	Liver Thyroid.	No
Dang et al. [24]	B6 WT Mice B6 ob/ob mice AML12 cells.	Animal model In vitro study.	Animal model 3 groups: B6 WT control B6 WT with obesity, high-fat diet. B6 ob/ob 3 months. In vitro study: Stimulus with exosomes from each of the groups. 48 h.	↑ 151-5p, miR-299a-5p, miR-135b-5p, miR-15b-3p, miR-306-5p, miR-431-5p, miR-449a-5p ↓ miR-141-3p, miR-351-5p, miR-874-3p	↓ STAT3, PPP1R3B, SLC2A2, GSK3B, PTEN, PIK3CB, IRS2, SLC2A4, RPS6KA1, PRKAA2, PTPN1, NFKB1, FOXO1, MAPK8, AKT3	Insulin resistance.	Adipose tissue Cell culture supernatant.	Yes
Pan et al. [25]	C57BL/6J WT mice with and without obesity KO mice for miR-34a.	Animal model.	WT and KO mice with obesity: high-fat diet. WT and KO mice without obesity: standard chow diet 16 weeks.	↑ miR-34a	↑ TNFα, IL6, IL1B, INOS, MCP1 ↓ FIZZ1, YM1, ARG1, IL10, KLF4	Proinflammatory cytokines Glucose intolerance Insulin resistance.	Adipose tissue Cell culture supernatant.	Yes
Castaño et al. [26]	C57BL/6J mice Group 1 with obesity Group 2 without obesity.	Animal model In vitro study.	High-fat diet Chow control diet 15 weeks.	↑ miR-192, miR-122, miR-27a ↓ miR-375	↓ PPARα, PPARγ, CD36, FADS1, PPARD, LIPE, PNPLA2 ↑ PLIN2, CPT1α, FGF21, CCL2, TNF	Adipogenesis Lipogenesis Fatty acid oxidation Inflammation Insulin resistance Dyslipidemia.	Plasma.	Yes
Liu et al. [27]	C57BL/6J mice 3T3-L1 adipocytes Human adipocytes: Group 1: nonobese BMI ≤ 25 kg/m^2^ Group 2: overweight and obese BMI > 25 kg/m^2^.	Animal model In vitro study.	Animal study: Group 1: Control diet with 15% L Group 2: High-fat diet with 45% L 8 weeks. In vitro study on 3T3-L1 adipocytes stimulated with or without 50 ng/mL TNFα.	↓ miR-1934	↑ TNF-α, IL-6, IL-1β, CD11c, MCP-1	Inflammatory state.	Adipose tissue Serum.	No
Clinical studies								
Heianza et al. [28]	Humans with overweight and obesity (BMI 32.7 ± 3.8 kg/m^2^).	Randomized controlled clinical trial (N = 495): Group 1 = 124 Group 2 = 113 Group 3 = 116 Group 4 = 92.	4 types of energy restricted diet: Group 1. Low fat and average protein (20% L, 15% P, 65% HC). Group 2. Low fat and high protein (20% L, 25% P, 55% HC). Group 3. High in fat and average protein (40% L, 15% P, 45% HC) Group 4. High fat and high protein (40% L, 25% P, 35% HC) + 90 min of moderate exercise a week 6 months.	↑ miR-128-1-5p	↓ LCT, R3HDM1, PRDM, PPARγ, C1A, PPARα	Energy expenditure Glucose metabolism Insulin resistance.	Plasma.	No
Cabiati et al. [29]	Adolescents with obesity BMI 29.5 ± 0.8 kg/m^2^ Adolescents without obesity BMI 21.1 ± 0.6 kg/m^2^.	Cross-sectional clinical study (N = 44), (22 per group).	No treatment.	↓ miR-223-5p, 33a-3p, miR-181a-5p, miR-199-5p	↓ CHD9, PTEN, MTMR12, TBL1X, CPOX, ACOT9	Lipid metabolism Inflammatory state Insulin resistance.	Plasma.	Yes
Zhang and Pan [30]	Adolescents with obesity and no non-alcoholic fatty liver disease Adolescents with obesity and non-alcoholic fatty liver disease.	Cross-sectional clinical trial (N = 10), (5 per group).	No treatment.	↑ miR-122-5p, miR-27a, miR-335-5p	↓ WNT10B, PPARγ, SREBP-1C	Hepatic lipid metabolism Adipocyte differentiation.	Serum.	Yes
Eirin et al. [31]	Vascular stromal adipose cells from abdominal subcutaneous fat tissue of two groups: Humans with obesity (BMI ≥ 30 kg/m^2^) Humans without obesity (BMI ≤ 25 kg/m^2^) Proximal tubule epithelial cells (HK2).	Cross-sectional clinical study In vitro study (N = 10), (5 per group).	HK2 stimulation: a model of ischemic kidney injury with TNF-α 10 ng/mL and antimycin A (AMA).	↑ miR-1291, miR-888-5p, miR-6892, miR-222-5p, miR-8072, miR-4757-5p, miR-769-5p, miR-4730 ↓ miR-346, miR-650, miR-634, miR-429, miR-136-3p, miR-222-3p, miR-124b-3p, miR-454-5p, miR-552, miR-208b-3p, miR-9-3p, miR-548-5p, miR-20a-5p, miR-545-5p, miR-455-3p, miR-146a-3p	↑ NFK-β, PP38, MAPK ↓ WNT1	MAPK pathway Apoptosis NF-κβ signaling pathway.	Cell culture supernatant.	Yes
Al-Rawaf [32]	Humans Adolescents Group 1: Obese (BMI 26.7 ± 2.91 kg/m^2^) Group 2: Overweight (BMI 21.9 ± 5.7 kg/m^2^) Group 3: Normal weight (BMI 17.4 ± 4.3 kg/m^2^).	Cross-sectional clinical trial (N = 250): Group 1 = 100 Group 2 = 100 Group 3 = 50.	No treatment.	↑ miR-142-3p, miR-140-5p, miR-22, miR-143, miR-130 ↓ miR-532-5p, miR-423-5p, miR-520c-3p, miR-146a, miR-15a	↓ P38, MAPK	Lipid metabolism.	Plasma.	No
Cannataro et al. [33]	Humans with obesity (BMI 46 ± 10 kg/m^2^) separated by sex.	Controlled clinical trial (N = 36): Women = 18 Men = 18.	Hypocaloric ketogenic diet with 2 phases: Phase 1: −300 kcal, 30 g HC, 57% L, 37% P. Phase 2: −200 kcal, 120 g HC, 44% L, 32% P 3 weeks each phase.	↑ miR-504-5p ↓ miR-Let7-5p, miR-143-3p, miR-30a-5p, miR-502-5p, miR-590-5p, miR-644a, miR-148b-3p, miR-26a-5p, miR-520, miR-548bmi-3p	↓ ALDH, CS, DLAT, GPI, HK, PFKM, BPGM, ACACA, CRKL, GYS, IRS2 Y 4, MAPK, NRAS, PHKA, PRKAA, PPARγ, AKT, HRAS, KRAS, ACSL, ALDH, MCAT, HADHA, HMGCS, LPL, RXRB, SCD, FADS, SLC, ATP, OX, NDUFA, NFKBIA, HIF, HSL, FHIT, TP53, VEGFA, MDM2, TFF1, TCEA1, DRD1	Glycolysis, gluconeogenesis and citrate cycle Insulin signaling pathway Fatty acid metabolism PPAR signaling pathway mTOR, amino acids and cytokine signaling.	Serum.	No
Tang et al. [34]	Humans: Group 1: adolescents with obesity (BMI 33.2 ± 4.23 kg/m^2^) Group 2: adolescents without obesity (BMI 23.21 ± 4.23 kg/m^2^).	Controlled clinical study (N = 67): Group 1 = 57 Group 2 = 10.	Group 1: Restrictive diet (20–25 Kcal/day) + exercise program of 50 min, 5 days a week. Group 2: Control diet + sedentary lifestyle 6 weeks.	↑ miR-126	↓ SPRED-1, PI3KR2, CXCL12	Angiogenesis PI3K-eNOs pathway.	Serum.	No
Parr et al. [35]	Humans: Overweight and obese (BMI 27–40 kg/m^2^).	Controlled clinical study (N = 89): Diet 1 = 32 Diet 2 = 29 Diet 3 control = 28.	Energy restriction diet (<250 Kcal) Diet 1: high protein and high HC (~30% P, 55% HC, 15% L) Diet 2: high protein and moderate HC (~30% P, 40% HC, 30% L) Diet 3 control: low protein and high HC (15% P, 55% CH, 30% L) + exercise with energy expenditure of ~250 Kcal 16 weeks.	↓ miR-221, miR-223, miR-140, miR-935, miR-448, miR-310, miR-263	↓ ARL15, CROT, CRTC3, FNDC5, SOCS7, STRADE, CYP7A1, PIK3R1, HDAC4, IGF1R, PLA2G6, SORBS, HMGB1, MEF2D, PHIP, PPARγ	Lipid metabolism and β fatty acid oxidation. Regulation of energy metabolism Insulin metabolism Low grade inflammation.	Serum.	No
Tabet et al. [36]	Humans: Group 1 High protein diet: Obesity (BMI 32.7 ± 4.2 kg/m^2^) Group 2 Normal-protein diet: Obesity (BMI 32.6 ± 4.4 kg/m^2^).	Controlled clinical trial (N = 47): Group 1 = 20 Group 2 = 27.	Group 1: High protein diet Group 2: Normal protein diet 12 weeks.	↑ miR-223	↓ GLUT4	Glucose metabolism.	Serum.	No
Ortega et al. [37]	Humans: BMI 30–35 kg/m^2^.	Clinical study First phase (N = 10) Second validation phase (N = 30).	Isocaloric diet: 55–60% HC, 15% P, <30% L (<10% saturated fats, 10-15% monounsaturated fats, 10% polyunsaturated fats; 5–8% omega-6, 1–2% omega-3) 15 g almonds and 15 g walnuts; for 8 weeks.	↑ miR-328, miR-330-3p, miR-221, miR-125-5p ↓ miR-192, miR-486-5p, miR-19b, miR-106a, miR-18a, miR-130b	Not mentioned	Inflammatory state Lipid metabolism.	Plasma.	No
Pescador et al. [38]	Humans: Group 1: Obesity BMI 42.7 ± 4.67 kg/m^2^ Group 2: Control BMI 22.7 ± 2.43 kg/m^2^ Group 3: DM2 BMI 24.8 ± 1.49 kg/m^2^ Group 4: OB-DM2 BMI 33.3 ± 3.86 kg/m^2^.	Cross-sectional clinical trial: Group 1 = 20 Group 2 = 20 Group 3 = 13 Group 4 = 16.	No treatment.	↑miR-138, miR-15b, miR-376a	↓ P85a, PIK3R1	Hepatic triglyceride storage Apoptosis Adipogenesis.	Serum.	No
Ortega et al. [39]	Humans: Group 1: Obesity BMI 42.9 ± 5.9 kg/m^2^ Group 2: Obesity BMI 32.4 ± 3.8 kg/m^2^.	Cross-sectional and longitudinal clinical trial First phase identification = 32 Group 1 = 6 Second phase validation = 102 Group 2 = 9.	Group 1: Bariatric surgery Group 2: Energy restrictive diet.	↑ miR-142-3p, miR-140-5p, miR-222 ↓ miR-221, miR-130b, miR-423-5p, miR-15a, miR-520-3p	↑ TGFRB1, LIFR, VEGFA	JAK-STAT and MAPK pathway Adipocyte development Energy expenditure Apoptosis Angiogenesis.	Plasma.	No

Abbreviations: VCAM1: Vascular cell adhesion molecule 1; ICAM1: Intercellular adhesion molecule 1; MCP1: C-C motif chemokine ligand 2; P65: RELA proto-oncogene; GANAB: glucosidase II alpha subunit; VCL: Vinculin; SYNPO2: Synaptopodin 2; TFRC: Transferrin receptor; WARS1: tryptophanyl-tRNA synthetase 1; PLEC: plectin; EEF2: eukaryotic translation elongation factor 2; MAP 1, 4: Microtubule associated protein; SND1: Staphylococcal nuclease and tudor domain containing 1; RPS15A: Ribosomal protein S15a; ZYX: Zyxin; CNDP2: Carnosine dipeptidase 2; ACSL1, 3, 4: Acyl-CoA synthetase long chain family member 1, 3, 4; ADIPOQ: Adiponectin; Akt: serine/threonine-protein kinase; ATP5A: ATP Synthase α-subunit; BMI: Body Mass Index; C/EBP: CCAAT/Enhancer Binding Protein; CHD9: Chromodomain Helicase DNA Binding Protein; CHUK: Conserved Helix-loop-helix Ubiquitous Kinase; CPOX: Coproporphyrinogen Oxidase; DIO1: Iodothyronine Deiodinase 1; FATP1: Fatty acid Transport Protein 1; FBXW7: F-Box And WD Repeat Domain Containing 7; FOXO1: forkhead box O1; G6PC: Glucose-6-Phosphatase Catalytic; GGT1: Gamma-Glutamyltransferase 1; GLUT4: Glucose Transporter Type 4; GPX7: glutathione peroxidase 7; HDL: high-density lipoproteins; HK3: Hexokinase 3; HSP60: heat shock protein 60; IL-4, 6, 13, 10: Interleukin 4, 6, 13, 10; Insig2: Insulin-induced Gene 2; IRS1: Insulin Receptor Substrate-1; JNK: c-Jun N-terminal Kinase; KLF4: Krueppel-like factor 4; LDL: low-density lipoproteins; lncRNAs: long noncoding RNAs; MAPK: Mitogen-Activated Protein Kinase; MTMR12: Myotubularin Related Protein 12; mTOR: Mechanistic Target of Rapamycin; NADK: Nicotinamide adenine dinucleotide kinase; NIS: iodine transporter protein; Nrf2: Nuclear factor erythroid 2-related factor 2; OXPHOS: Oxidative Phosphorylation System; Pcsk9: Proprotein Convertase Subtilisin/Kexin Type 9; Per3: period circadian regulator 3; PFKP: Phosphofructokinase; PGC-1a: Peroxisome Proliferator-Activated Receptor γ Coactivator 1 α; PKM2: Pyruvate kinase M2; PPARα: Peroxisome Proliferator-Activated Receptor α; PPARγ: Peroxisome Proliferators–Activated Receptor γ; PTEN: Phosphatase and Tensin Homologue; PTEN: phosphatase and tensin homologue; PTGDS: Prostaglandin D2 Synthase; SLC2A5: Solute Carrier Family 2 Member 5; Socs1/3: Suppressor of Cytokine Signalling 1 y 3; SOD2: superoxide dismutase 2; SREBF1: Sterol regulatory element-binding transcription factor 1; SREBP-1C: Sterol Regulatory Element-binding Protein 1; STAT3: Signal Transducer and Activator of Transcription 3; TBL1X: Transducin Beta Like 1 X-Linked; TET3: Tet methyl cytosine dioxygenase; TGF-β: Transforming Growth Factor-Beta; TLR4: Toll-Like Receptor 4; TMEM173: Transmembrane Protein 173; TRB: T-Cell Receptor Beta Locus; UCP1: Uncoupling Protein 1; VDR: Vitamin D receptor; WNT10B: Wnt Family Member 10B.

**Table 2:** MiRNAs, exosomal miRNAs, insulin resistance, and diet.

**Author**	**Study Population**	**Study Design**	**Dietary Intervention/Treatment**	**miRNAs Regulated**	**Significant Target Genes**	**Metabolic Effect**	**Tissue**	**Exosomes**
In vitro studies								
Li et al. [40]	Mouse pancreatic cells MIN6 3T3-L1 cells C57BL/6J WT control mice C57BL/6J Lep ob (ob) mice Humans with DM2 with overweight and obesity (BMI > 25 kg/m^2^).	In vitro study Animal model Cross-sectional clinical study.	Animal model High fat diet (20% CH, 20% P, 60% L) + injections with exosomes from control mice 6 weeks.	↑ miR-29a, miR-29b, miR-29c	↓ MCTL, STXLA, PI3KRL, P85α	Hepatic glucose production Glucose homeostasis	Cell culture supernatant Plasma.	Yes
Wen et al. [41]	3T3-L1 adipocytes Rat ventricular myocytes.	In vitro study.	Stimulus with palmitate Stimulus with insulin.	↑ miR-802-5p	↓ HSP60	Oxidative stress Insulin resistance	Cell culture supernatant	Yes
Tian et al. [42]	Mouse macrophages RAW264.7 3T3-L1 adipocytes C57BL/6J WT mice C56BL/6J KO for miR-210-/- mice.	In vitro study Animal model.	In vitro study: Stimulus with exosomes (2 µg) of macrophages 24 h. Animal model 3 groups: WT control normal diet 2. WT with obesity, high fat diet 3. KO with obesity and diabetes high fat diet + streptozotocin 7 weeks.	↑ miR-210	↓ NDUFA4	Mitochondrial dysfunction Insulin resistance	Cell culture supernatant Serum.	Yes
Su et al. [43]	Bone marrow mesenchymal stem cells from 3- and 18-month-old mice 3T3-L1 adipocytes C2C12 myocytes Primary hepatocytes C57BL/6J WT mice.	In vitro study Animal model.	In vitro study: Stimulation with bone marrow mesenchymal stem cell exosomes from 3- and 18-month-old rats to adipocytes, myocytes and hepatocytes. 12 h. Animal model: Exosomes from bone marrow mesenchymal stem cells of 3- and 18-month-old rats 7 days.	↑ miR-29b-3p, miR-17-50, miR-762, miR-465b-5p, miR-221-3p, miR-6409, miR-151-5p, miR-191-5p, miR-99b-5p, miR-7118-5p, miR-6347, miR-290b-3p, miR-let-7a-5p, miR-3170-5p, miR486-5p, miR-107-3p, miR-486-5p, miR-107-3p, miR-24-3p, miR-1931, miR-92b-3p, miR-20a-5p, miR-6968-5p ↓ miR-190a-3p, miR-702-5p	↓ SIRT1	Insulin resistance	Cell culture supernatant.	Yes
Ying et al. [16]	3T3-L1 adipocytes WT mice without obesity WT mice with obesity KO mice for miR-155 L6 myocytes cells.	In vivo study Animal model.	Stimulus with macrophages transfected with miR-223 Treatment with macrophage exosomes from adipose tissue. KO mice for miR-155: High fat diet 20 weeks.	↑ miR-155, miR-181a, miR-149, miR-210, miR-1945 ↓ miR-3968, miR-692, miR-365, miR-7070, miR-5098, miR-1928, miR-690, miR-7054, miR-682, miR-1946, miR-511	↓ PPARγ, GLUT4	Insulin resistance Glucose homeostasis	Cell culture supernatant.	Yes
In vivo studies in animal models								
Xiong et al. [44]	Small rats by gestational age (SGA) Control term rats Primary hepatocytes 3T3-L1 adipocytes L6 myocytes.	Animal model In vitro study.	Pregnant rats Control group: Standard diet ad libitum. SGA rats group: Energy restriction (50% of normal intake).	↑ miR-210	↓ SIDT2	Insulin resistance Autophagy Lipid metabolism Inflammation	Cell culture supernatant.	Yes
Hong et al. [45]	Polycystic ovary syndrome mice with insulin resistance 3T3-L1 adipocytes.	Animal model In vitro study.	High-fat diet + dehydroepiandrosterone.	↓ miR-20b-5p, miR-106a-5p	Not mentioned	Lipid metabolism Adipocyte differentiation	Serum.	Yes
Wang et al. [46]	Mice with DM2 Control mice without DM2 3T3-L1 adipocytes AML12 cells.	Animal model In vitro study.	DM2 model: High-fat diet + streptozocin. Non-DM2 model: Normal Chow diet. 3T3-L1 and AML12 stimulation: NK cell exosomes from high-fat diet and control diet.	↑ miR-1906, miR-696, miR-7649-5p, miR-1187, miR-129b-5p, miR1249-3p ↓ miR-1249, miR-296-5p, miR292-3p, miR-290b-3p, miR-6866	↓ PAKT, PPARγ, GLUT4	Insulin sensitivity Inflammation	Cell culture supernatant.	Yes
Li et al. [47]	WT C57BL/6K mice with and without obesity Hep1-6 cells HEK293 cells.	Animal model In vitro study.	Mice with obesity: High fat diet (45% of L). Mice without obesity: Normal diet Chow. HEP1-6 stimulation: miR-143-5p mimics. HEK293 cell stimulation: miR-143-5p mimics and pmirGLO-MKP5.	↑ miR-143-5p	↓ DUSP10	Decreased AKT and GSK phosphorylation Glycogen synthesis	Culture medium supernatant.	Yes
Jalabert et al. [48]	Leptin-deficient C57BL/6 mice (ob/ob) C57BL/6 WT control mice 3T3-L1 adipocytes C2C12 muscle cells.	Animal model In vitro study.	Normal chow diet (57% HC, 25% L, 18% P) 12 weeks 3T3-L1 cells incubated with exosomes from animal models C2C12 cells incubated with exosomes from animal models.	↑ miR-1a-3p, miR-101a-3p, miR-340-5p, miR-434-5p, miR-106-b, miR-146a-5p, miR-24-3p, miR-200-3p, miR-203-3p ↓ miR-224-5p, miR-29b-5p, miR-495-3p, miR-434-3p, miR-299a-5p	↓ PPARγ, PPARα, CPT1, CPT2, CD36, ABCA1, HMGCR, CIDEC, FABP4, INSR, IGF1R	Lipid metabolism TNF-β pathway WNT pathway Proteolysis Thyroid hormone signaling Adrenergic signaling in muscle Insulin resistance	Cell culture supernatant.	Yes
Sun et al. [49]	db/m mice db/db mice Primary pancreatic islets.	Animal model In vitro study.	Normal diet control High fat diet 4 weeks.	↑ miR-29a-5p, miR-30a-5p, miR378a-3p, miR-203-3p, miR-486a-3p, miR-206-3p, miR-1a-3p, miR-30a-3p, miR-145a-5p, miR-192-5p, miR-146a-5p ↓ miR-Let-7-5p, miR-204-3p, miR-3473b, miR-193b-5p, miR-574-5p, miR-423-5p, miR-128-3p, miR-760-3p, miR-505-5p	↓ TRAF3, CXCL10, AHSG, P2RX1, KNG1, NOS2, CXCL17	Glucose intolerance Insulin resistance Inflammatory response	Cell culture supernatant.	Yes
Li et al. [50]	C57BL/6J mice With obesity-related insulin resistance Without obesity or insulin resistance.	Animal model	High fat diet Normal chow diet (control) 8 weeks.	↑ miR-222	↓ IRS1	Insulin resistance	Serum Gonadal adipose tissue.	Yes
Liu et al. [51]	C56BL/6J Mice With obesity Without obesity 3T3-L1 adipose tissue L6 myocytes Primary hepatocytes.	Animal model In vitro study.	Mice with obesity: High-fat diet (60% L, 20% P, 20% HC). Mice without obesity: Normal chow diet (control) 3 months. Stimulus in vitro study: miR-29a mimic.	↑ miR-29a	↓ PPARγ	Insulin resistance	Serum Adipose tissue.	Yes
Jalabert et al. [52]	C57BL/6J mice Primary pancreatic islets MIN6B1 cell line C2C12 myoblasts 3T3-L1 preadipocytes.	Animal model In vitro study.	Animal model: Group 1. Standard chow diet (57% HC, 25% L, 18% P). Group 2. Standard chow diet enriched with 20% palm oil. 16 weeks. In vitro study: Stimulation with skeletal muscle exosomes from animal model (group 1 and group 2).	miR-146a, miR-92a, miR-16	↓ PTCH1, HMGA1, WDR26, PDPK1, MXD1, RAP1A, RHOB, CCNE2, GPR137C, ATXN3	Adipogenesis and cell differentiation Cell cycle MAPK signaling pathway PI3K/Akt signaling pathway Insulin resistance	Tissue (muscle conditioning medium).	Yes
Clinical studies								
Infante-Menéndez et al. [53]	Humans with non-alcoholic hepatic steatosis Humans without non-alcoholic hepatic steatosis ApoE-/- mice with hepatic steatosis C57BL/6J WT mice without hepatic steatosis Huh7 cells.	Cross-sectional clinical study: Humans with non-alcoholic hepatic steatosis = 30 Humans without non-alcoholic hepatic steatosis = 21 Animal model In vitro study.	Animal model Steatosis mice: high fat diet Control mice: standard diet 13 weeks Huh7 cell stimulation with oleic acid and palmitic acid.	↑ miR-Let-7d, miR-34-5p ↓ miR-26b-5p,	↓ AKT, IGF1, INSR, IGF1R	Insulin resistance Cell proliferation Cell differentiation	Plasma Liver tissue.	Yes
Ye et al. [54]	Humans with newly diagnosed DM2 Healthy human controls HepG2 cells.	Prospective clinical study: Humans with newly diagnosed DM2 = 9 Healthy human controls = 9 Validation phase = 161 In vitro study.	In vitro study: Zinc sulfate stimulation from 0 to 140 µM. 24 h, then insulin stimulation Transfected with miR-144-3p mimic, 200 nM miR-144-3p inhibitor or negative control with Lipofectamine + zinc sulfate 18 h.	↑ miR-215-5p, miR-144-3p	↓ NRF2	Insulin resistance Oxidative stress TGF-β pathway	Plasma.	No
Ali et al. [55]	Humans with DM2 Humans with prediabetes Healthy humans (control).	Cross-sectional clinical study: Humans with DM2 = 66 Humans with prediabetes = 49 Healthy humans (control) = 45.	No treatment.	↑ miR-611, miR-5192, miR-1976	↑ CHUK, TMEM173	Inflammation Insulin resistance INF pathway activation	Serum.	No
Brandão-Lima et al. [56]	Humans with metabolic syndrome.	Cross-sectional clinical study (N = 192): Men = 87 Women = 105.	No treatment.	↑ miR-122, miR-Let-7c, miR-15a, miR-222, miR-146a, miR-miR-30d ↓ miR-139, miR-16, miR-363, miR-486	↓ IGF2BP, RFX6, IL10, CCL3, PDK4, SIRT4, AKAT3, VEGFA, IKBKB, CDK8, FOXO1, BCL2, MARK1, UCP3, PPARγ, C1A ↑ SOCS1/4, PIK3R1, CDK8, MAP3K2, IGF1, TRAF6, PRKAR2A	Inflammatory state Cytokine signaling PKA activation Stress response Insulin signaling Energy metabolism	Plasma.	Yes
Byun et al. [57]	Humans with overweight BMI 26.5 ± 2.9 kg/m^2^ and DM2 Humans without overweight and DM2 C57BL/6 mice Submandibular lymph node cells.	Cross-sectional clinical study (N = 60): Overweight humans BMI 26.5 ± 2.9 kg/m^2^ and DM2 = 30 Humans without overweight or DM2 = 30 Animal model In vitro study.	Animal model: Group 1: high fat diet Group 2: normal chow diet 12 weeks. In vitro study: Phorbol myristate acetate.	↑ miR-92-3p, miR-25-3p, miR-1290, miR-576-5p, miR-221-3p, miR-205-5p, miR-7847-3p, miR-320b, miR-25-3p, miR-451a, miR-130b-3p, miR-210-3p, miR-874-3p, miR-486-5p	↑ IL17A, IL17F ↓ RORC	Cell differentiation Inflammatory response Insulin resistance	Saliva Cell culture supernatant.	Yes
Mantilla-Escalante et al. [58]	Overweight and obese older adults BMI 29 ± 4 kg/m^2^.	Longitudinal clinical study (N = 150): Group 1 = 50 Group 2 = 50 Group 3 = 50.	Group 1: Mediterranean diet + extra virgin olive oil ≥ 4 tablespoons per day. Group 2: Mediterranean diet + nuts ≥ 3 servings per day. Group 3: Low-fat diet Duration: 1 year.	↓ miR-222-3p, mir-185-5p, miR-27a-3p, miR-21-5p, miR-29c-3p, miR-34b-5p, miR-320b, miR-107, miR-20b-5p, miR-20a-3p, miR-1246, miR-106a-5p, miR-23a-3p, miR-28-5p, miR-215, miR-21, miR-34, miR-103, miR-151, miR-22, miR-671-5p, miR-200c-3p, miR-193a-3p, miR-381-3p, miR-10b-3p, miR-24-3p, miR-122-5p, miR-16-5p, miR-28-5p, miR-195-50, miR-15a-5p, miR-26a-5p ↑ miR-215-5p, miR_369-3p, miR-10a-5p, miR-210-3p, miR-215-5p	↓ AKT1, CDK1, MYC, BHLH, PTEN, PLK1, TP53, MAPK1, CCND1, AMPK, FOXO, P53, HIF1	Insulin resistance Tumor suppression Cell proliferation and growth	Plasma.	Yes
Sardu et al. [59]	Overweight and obese humans with carotid artery stenosis Group 1: normal glycaemia Group 2: prediabetes without metformin treatment Group 3: prediabetes with metformin treatment.	Longitudinal clinical study (N = 234): Group 1 = 125 Group 2 = 73 Group 3 = 36.	Carotid revascularization surgery.	↑ miR-24, miR-27, miR-100, miR-126, miR-133	Not mentioned	Glucose metabolism Atherosclerosis	Serum.	Yes
Yu et al. [60]	Children with and without obesity Mice with obesity KO for miR-27a db/db mice C2C12 cells.	Cross-sectional clinical study (N = 90): Children with obesity = 45 Children without obesity = 45 Animal model In vitro study.	Animal model: High-fat diet Low fat diet 12 weeks. In vitro study: Stimulus with palmitate-treated 3T3-L1 adipocyte culture medium.	↑ miR-27a	↓ IRS1, GLUT4, PPARγ	Insulin resistance	Serum.	Yes

Abbreviations: ACSL1, 3, 4: Acyl-CoA synthetase long chain family member 1, 3, 4; ADIPOQ: Adiponectin; Akt: serine/threonine-protein kinase; ATP5A: ATP Synthase α-subunit; ATXN3: ataxin-3; BMI: Body Mass Index; C/EBP: CCAAT/Enhancer Binding Protein; CCNE2: cyclin E2; CHD9: Chromodomain Helicase DNA Binding Protein; CHUK: Conserved Helix-loop-helix Ubiquitous Kinase; CPOX: Coproporphyrinogen Oxidase; DIO1: Iodothyronine Deiodinase 1; DUSP10: dual specificity phosphatase 10; FATP1: Fatty acid Transport Protein 1; FBXW7: F-Box And WD Repeat Domain Containing 7; FOXO1: forkhead box O1; G6PC: Glucose-6-Phosphatase Catalytic; GGT1: Gamma-Glutamyltransferase 1; GLUT4: Glucose Transporter Type 4; GPR137C: G protein-coupled receptor 137C; GPX7: glutathione peroxidase 7; HK3: Hexokinase 3; HMGA1: high mobility group AT-hook 1; HSP60: heat shock protein 60; IL-4, 6, 13, 10, 17: Interleukin 4, 6, 13, 10, 17; Insig2: Insulin-induced Gene 2; IRS1: Insulin Receptor Substrate-1; JNK: c-Jun N-terminal Kinase; KLF4: Krueppel-like factor 4; LDL: low-density lipoproteins HDL: high-density lipoproteins; lncRNAs: long noncoding RNAs; MAPK: Mitogen-Activated Protein Kinase; MTMR12: Myotubularin Related Protein 12; mTOR: Mechanistic Target of Rapamycin; MXD1: MAX dimerization protein 1; NADK: Nicotinamide adenine dinucleotide kinase; NIS: iodine transporter protein; Nrf2: Nuclear factor erythroid 2-related factor 2; OXPHOS: Oxidative Phosphorylation System; Pcsk9: Proprotein Convertase Subtilisin/Kexin Type 9; PDPK1: 3-phosphoinositide dependent protein kinase 1; Per3: period circadian regulator 3; PFKP: Phosphofructokinase; PGC-1a: Peroxisome Proliferator-Activated Receptor γ Coactivator 1 α; PKM2: Pyruvate kinase M2; PPARs: Peroxisome Proliferator-activated Receptors; PPARγ: Peroxisome Proliferators–Activated Receptor γ; PTCH1: patched 1; PTEN: Phosphatase and Tensin Homologue; PTEN: phosphatase and tensin homologue; PTGDS: Prostaglandin D2 Synthase; RAP1A: member of RAS oncogene family; RHOB: Ras homologue family member B; SIDT2: SID1 transmembrane family member 2; SLC2A5: Solute Carrier Family 2 Member 5; Socs1/3: Suppressor of Cytokine Signalling 1 y 3; SOD2: superoxide dismutase 2; SREBF1: Sterol regulatory element-binding transcription factor 1; SREBP-1C: Sterol Regulatory Element-binding Protein 1; STAT3: Signal Transducer and Activator of Transcription 3; TBL1X: Transducin Beta Like 1 X-Linked; TET3: Tet methylcytosine dioxygenase; TGF-β: Transforming Growth Factor-Beta; TLR4: Toll-Like Receptor 4; TMEM173: Transmembrane Protein 173; TRB: T-Cell Receptor Beta Locus; UCP1: Uncoupling Protein 1; VDR: Vitamin D receptor; WDR26: WD repeat domain 26; WNT10B: Wnt Family Member 10B.
